# The Impact of Community Generated PPE During the SARS-COV-2 Pandemic in Southeast Alabama and Gulfport Mississippi

**DOI:** 10.3389/frhs.2021.786062

**Published:** 2021-12-15

**Authors:** Benjamin Buttars, Leigh Fountain, Joseph Goodwin, Jessica McLean, John Olsen, Trey Hatton, William C. Grant, Audrey Vasauskas, Caleb Hamilton, Martin Clemmons

**Affiliations:** ^1^Department of Research, Alabama College of Osteopathic Medicine, Dothan, AL, United States; ^2^Alabama College of Osteopathic Medicine and Infirmary Health Department of Internal Medicine Residency, Fairhope, AL, United States; ^3^Department of Biology, Troy University, Troy, AL, United States

**Keywords:** pandemic, personal protective equipment, SARS-CoV-2, community, local

## Abstract

**Background:** The early stages of the SARS-COV-2 pandemic left many hospital systems devoid of personal protective equipment. Community-driven groups manufactured Personal Protective Equipment (PPE) as a form of temporary replacement until supply could increase to frontline healthcare workers. The purpose of this study was to survey hospital systems in Alabama and Mississippi who requested and received PPE to determine recipient opinions concerning community involvement.

**Methods:** A 15-question Qualtrics survey was distributed to hospital systems who requested and received community-generated PPE (CGPPE) from the group known as Alabama Fighting COVID. 275 responses were gathered over a period of 6 months.

**Results:** Survey data showed that most respondents from healthcare and healthcare-associated professions responded that wearing community generated personal protective equipment provided them with the perception of added protection (55.31% of participants selected either “Agree” or “Strongly Agree”), and that it improved their outlook and desire to work during the pandemic (51.77% of participants selected either “Agree” or “Strongly Agree”).

**Conclusions:** Most respondents reported that wearing community generated personal protective equipment not only provided them with the perception of added protection, but that it improved their outlook and desire to work during the pandemic. With these responses in mind, our study raises questions concerning whether local CGPPE distribution could improve well-ness outcomes of healthcare workers (HCWs) not only in relation to decreased viral transmission, but also in favorable psychosocial health assessments. Further implications for research concerning community involvement during future medical crises are indicated, especially with the current rise of the delta variant strain.

## Introduction

The initial SARS COVID pandemic introduced new challenges in the world health community as the viral illness passed from person to person at an alarming rate. Among many others, one immediate and crippling difficulty was the shortage of personal protective equipment (PPE) (1). Many frontline healthcare workers quickly ran out of ways to decrease risk of infection *via* exposure. Community groups began coordinated efforts to supply the healthcare field with substitute forms of protective equipment while the normal supply chain caught up with the sudden and drastic worldwide demand for such things as masks and face shields. Community generated personal protective equipment (CGPPE) came in several forms, including, but not limited to, hand-sewn masks, 3d printed face shields and “ear savers” (1). The term “ear savers” refers to small clips used to fasten mask elastic behind the neck instead of placing them on the ears. These were distributed in hospitals, clinics, dental offices, and other essential “front line” points of care (1).

Social support may help healthcare workers (HCWs) cope with the heightened stress of such crises. A perceived lack of social support is a contributing factor to both HCW stress and burnout (2, 3). Healthcare workers who perceive significant stress often experience decreased job satisfaction and a resulting higher rate of turnover (4). Heightened HCW stress has been shown to increase the incidence of reported minor medical mistakes (5). Additionally, the impact of stress and burnout in HCWs may extend beyond the workplace *via* increased risks of developing hypertension, increased left ventricular mass, and increased diastolic blood pressure (6).

Recent studies have evaluated the toll that COVID has taken on the psyche of HCWs and have collectively demonstrated heightened psychological distress, depression, and anxiety in those participating directly in patient care (7–9). Mediavilla et al., stated that insufficient PPE availability was shown to be a significant modifiable work-related stressor associated with detrimental mental health impact among healthcare employees (10). Collectively, HCWs endure an emotional burden during disease outbreaks that should be addressed. This study proposes two possible solutions to partially alleviate said burden: access to PPE and social support.

The purpose of this initial study was to determine if individuals working in healthcare acknowledged benefit, whether intrinsic or extrinsic, from CGPPE in order to promote discussion about potential pathways of distribution should future shortages arise.

## Methods

An original survey developed by the research team consisting of 15 questions was distributed *via* the Qualtrics platform by email to hospital systems, local clinics, and nursing homes in the Dothan, AL; Birmingham, AL; and Gulfport, MS areas (see Appendix for the complete survey). Subjects were selected based on a request list for CGPPE through the Wiregrass COVID Coalition and Birmingham Fighting COVID groups. Administration from the clinics and hospitals were contacted to request permission to send a survey link to employees who had received CGPPE. The three hospital systems surveyed requested that all employee information and their names remain anonymous. In total, three hospitals, five private clinics, five nursing home/assisted living centers, and two dental clinics participated. Only hospitals who had requested and received CGPPE were contacted for potential survey participation. Not all who requested CGPPE elected to participate. The survey was designed to measure attitudes and perceptions concerning CGPPE. This study was approved by the Alabama College of Osteopathic Medicine (ACOM) Institutional Review Board. All participants consented to the study and responses were collected anonymously. Data were compiled, excluding any missing question responses and data points, and the descriptive statistics were obtained from the Qualtrics platform. The data were then exported to SPSS Statistics for Windows, Version 27.0 (SPSS Inc., Chicago, IL, USA), and associations between Likert scale responses were assessed *via* Spearman Correlations. One survey respondent stated that they never used CGPPE and as such, they were excluded from responses involving their opinion of the CGPPE. All other responses were included.

## Results

The survey was open for a period of six (6) months, from July 1, 2020 to December 31, 2020. Responses to questions regarding work setting, hospital department, and work title are displayed in Table 1.

**Table 1:** Demographic and workplace factors.

	**Number of responses**	**Percentage of responses**
**Work setting**		
Hospital	214	79.95%
Independent clinic	48	17.92%
Home health facility	6	2.24%
**Hospital department**		
Non-ICU/Med-surg floor	52	19.92%
Outpatient	44	16.86%
ICU/CCU	18	6.9%
ER	15	5.75%
Labor and delivery	10	3.83%
Neonatal ICU	4	1.53%
Administrative/Other	118	45.21%
**Work title**		
BSN/RN/LPN	82	31.54%
Support associates (reception, med tech, X ray tech, CNA, etc.)	69	26.54%
Healthcare administration	21	8.08%
Physicians	9	3.46%
Physical/occupational/speech therapy	9	3.46%
Pharmacists	6	2.31%
Physician's assistant/nurse practitioner	6	2.31%
Respiratory therapy	2	0.77%
Other	56	21.54%

*ICU, Intensive Care Unit; Med-Surg, Medical-Surgical; CCU, Critical Care Unit; ER, Emergency Room; BSN, Bachelors of Science in Nursing; RN, Registered Nurse; LPN, Licensed Practical Nurse; Med, Medical; Tech, Technician; CAN, Certified Nursing Assistant*.

Some of those who answered “other” with specifications such as registered nurse, pharmacy technician, gastrointestinal technician, and “RN on a COVID floor” were categorized appropriately and subtracted from the total number of “other” answers.

When asked what PPE was provided by their organizations, with the ability to choose more than one option: 206 answered N95 Masks or other masks, 116 answered Shields, 104 answered Sterile Gowns, 110 answered Surgical Gowns, and 69 answered Other and specified the PPE provided. The answers from the Other (Please Specify) were categorized into the previous categories when applicable, but the total number of Other answers was not changed. Further breakdown of the Other (Please Specify) answers: 11 answered various gowns, seven answered goggles, four answered gloves, four answered none, two answered lab coats, one answered hand sanitizer, one answered a sterile pack for the ebola crisis, and one answered cavi wipes. Responses to Likert-style and multiple-choice questions are provided in Table 2.

**Table 2:** Survey questions and responses.

**Question**	**Response**				
	**Strongly agree**	**Agree**	**Neither agree nor disagree**	**Disagree**	**Strongly disagree**
Does wearing the CGPPE provide you with the perception of added protection?	46 (20.35%)	79 (34.96%)	59 (26.11%)	24 (10.62%)	18 (7.96%)
Are you more likely to support the use/production of CGPPE because they are made locally?	53 (23.45%)	70 (30.97%)	70 (30.97%)	18 (7.96%)	15 (6.64%)
Wearing CGPPE improved my outlook toward working during the pandemic.	48 (21.24%)	69 (30.53%)	62 (27.43%)	30 (13.27%)	17 (7.52%)
Using CGPPE increased my desire to work during the time of the Pandemic.	32 (14.16%)	49 (21.68%)	84 (37.17%)	39 (17.26%)	22 (9.73%)
I would accept and use CGPPE in a future situation where there is an equipment shortage.	80 (35.40%)	105 (46.46%)	24 (10.62%)	8 (3.54%)	9 (3.98%)
I personally feel that my administration supported my well-being and safety during the COVID pandemic.	86 (38.05%)	82 (36.28%)	31 (13.72%)	15 (6.64%)	12 (5.31%)
I personally fell that my community supported my well-being and safety during the COVID pandemic.	79 (34.96%)	92 (40.71%)	30 (13.27%)	18 (7.96%)	7 (3.10%)
	**Increased**	**Decreased**	**Unchanged**		
Have there been changes in the volume/amount of PPE supplied by your organization since the start of the COVID crisis?	*137 (54.15%)*	*70 (27.67%)*	*46 (18.18%)*		
	**Yes**	**No**			
Have there been changes to the type of the PPE supplied by your organization since the start of the COVID crisis?	213 (82.24%)	46 (17.76%)			

**Figure 1:**
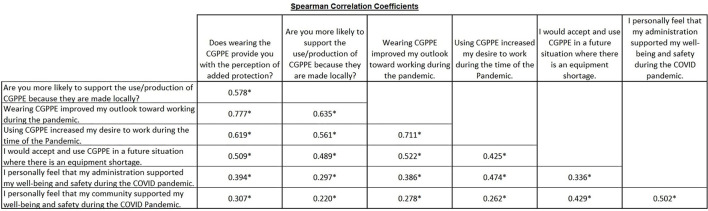
Associations between CGPPE and participant perception of safety and support. To explore the associations between the participants' perception of community contribution of PPE, personal safety, and the support of their employer and the community, Spearman Correlations were performed between the responses to the indicated Likert scale survey questions. Numeric values in the figure are representative of the Spearman correlation coefficient (r), with asterisks indicating significance (*p* < 0.05, two-tailed).

A Spearman Correlation was deployed (Figure 1) with the ordinal variable of the Likert Scale (1 = Strongly Agree, 2 = Agree, 3 = Neither Agree nor Disagree, 4 = Disagree, 5 = Strongly Disagree) based on the questions in the Appendix. There was a significant positive correlation (*p* < 0.05) between all of these questions with the lowest *r*- value being 0.220 and the highest *r*- value being 0.777.

## Discussion

In the middle of the COVID-19 pandemic, HCWs across the United States were expected to use PPE past manufacturers' recommendations. This obligation was due to the sudden and drastic increase in demand which resulted in an overly burdened readily available supply chain. As United States health systems struggled to manage the crisis in early 2020, the Centers for Disease Control and Prevention (CDC) recommended, among other measures, the creation of “home” PPE supplies (1, 11). Distribution of locally produced CGPPE filled this niche in the medical communities across Alabama and Mississippi. The cross-sectional study of 275 survey responses who received CGPPE provided information on the availability and impact of both community and administration-acquired PPE. 55.31 percent of respondents from healthcare and healthcare-associated professions either agreed or strong agreed that wearing community generated personal protective equipment provided them with the perception of added protection, and 51.77% of respondents indicated that it improved their outlook and desire to work during the pandemic.

To determine whether there were any associations between factors pertaining to the participants use and outlook regarding the manufactured PPE and their perception of well-being and safety, Liker scale responses were assessed *via* Spearman Correlations. This study demonstrated strong correlations between several separate factors (Figure 1). The strongest correlation was between respondents who positively ranked that wearing CGPPE improved their outlook toward working during the pandemic and those who positively ranked that wearing PPE provided them with the perception of added protection (*r* = +0.777). These HCWs may have experienced improved outlooks as a result of the extra protection they believed was occurring with a greater PPE availability provided by CGPPE. This could open into discussion on how perceived safety may create an emotional benefit that improves HCW outlooks during times of great stress. Furthermore, the respondents who indicated an improved outlook toward working also strongly correlated with being more likely to support CGPPE use and production because it was made locally (*r* = +0.635). Perhaps these individuals regarded local CGPPE donations as a form of appreciative support from their community for the work they were undertaking.

Similar forms of morale improvement in HCWs have been demonstrated in prior studies such as Grant et al., which showed significant increases in self-reported positive behavioral outcomes and motivation from healthcare employees after implementing public recognition and enhanced teamwork as a rewards system. These improvements were particularly notable in frontline HCWs when compared to workers in supervision roles and particularly in resource-constrained settings (12).

In this study, if the local community who produces CGPPE is viewed as an extension of a healthcare team and the generation of CGPPE as a form of public recognition, then findings complement those produced by Grant et al., The use of such rewards as tools for positive reinforcement could direct future guidelines regarding employee morale-especially under tight resource constraints like those seen in the COVID-19 pandemic. Nevertheless, further discussion needs to be made.

Respondents who positively ranked that they would accept CGPPE during future equipment shortages demonstrated the strongest correlation with CGPPE providing an improved perception of added protection (*r* = +0.509). This may signal that, above all else, the value of CGPPE to HCWs lies in its potential for protection. This may seem obvious since the main job of PPE is, inherently, to protect the user. However, it paints a picture of the other rewards of CGPPE, like improved outlook and desire to work, as potentially less determined by the CGPPE itself but rather as a product of an increase in perceived safety. With this in mind, future studies on HCW responses to separate types of CGPPE may provide greater insight into which forms of protection provide the greatest sense of safety to HCWs. And, to that end, this may guide which forms of CGPPE should be produced in the greatest amounts in order to garner the most positive results in HCWs in terms of outlook, desire to work, and perceived safety. While these specific answers are dependent on future studies to elucidate, what is already clear from the results is that HCWs do gain some benefits from CGPPE whether they be somatic, psychosomatic, and/or functional in terms of an improved barrier to disease spread.

## Implications for Research

Health care disasters have shown numerous implications on the health and well-ness of both individuals and communities (13). In particular, HCWs are uniquely vulnerable to adverse psychiatric stressors of public health crises due to their inherent risk of disease contraction and greater pressure to allocate scant resources such as PPE which directly impact the survival outcomes of their patients (14). Given that the majority of respondents in this survey noted an improved outlook with the use of CGPPE, a study into the correlation between CGPPE and improved psychosocial outcomes for healthcare professionals could inform future supply decisions.

SARS-CoV-2 is spread through viral travel in airborne particles, respiratory droplets, and on surfaces as fomites (15, 16). To extend the longevity of limited PPE supplies, HCWs have been pushed to reuse protective equipment without adequate peer-reviewed protocols on their efficacy. In an ideal world without limit on supply, most commercial PPE is designed for single-use and typically single-patient encounters. Under the CDC guidelines for crisis conditions, defined as “when supplies cannot meet the facility's current or anticipated PPE utilization rate,” HCWs are advised to both use PPE “beyond manufacturer designated shelf life” and “implement limited re-use” (11). Furthermore, when neither N95 respirators nor facemasks are available, HCWs under crisis guidelines should opt for a face shield that reaches to the chin and sides of face. These methods are backed by limited research and, where research does exist, it highlights the unsustainable nature of reusing single-use, disposable PPE. For instance, changes in the shape of reused N95 masks have adverse impacts on the integrity of the seal against the user's face, which is critical when dealing with SARS-CoV-2, a virus of around 0.05–0.2 μm (17, 18). In one study examining repeated donning and doffing of fitted N95 respirators, 48% percent of HCWs failed their fit test over the course of five repeat-uses. The greatest difference in seal was between the first and second uses (19). Single-use commercial PPE also exhibit a limited ability to retain structural integrity and filtration capacity as a result of decontamination treatments like autoclaving, chemical treatments, and UV light (20). Additionally, the use of combination PPE methods, such as sterile face shields and surgical masks, vs. re-used N95 masks have not been rigorously studied with SARS-CoV-2 (20). In the context of this research, further studies would need to be conducted into whether increased turnover of combination CGPPE provides effective protection when compared to long-term reuse of disposable medical-grade, administration-provided PPE.

## Strengths and Limitations

This study has multiple strengths. The scope of respondents crossed >8 occupational titles within the healthcare community, seven noted departments, and various patient care settings from inpatient to clinic and extended care facilities. Respondents also varied in their ages over a broad range from early twenties to over fifty years old.

This study also has limitations. While over 60,000 face shields were distributed in the greater south Alabama area, this study only samples 275 responses. To increase the power of future studies, a more robust quantity of responses is needed. Additionally, to maintain anonymity of the workers, the hospital systems requested to control distribution of the survey. The estimated response rate is 3.72% from a calculation of total employees at each facility with responses. It is unlikely that the survey reached every employee and therefore this calculation is a very low estimate.

Moreover, these inferences are based on a cross-sectional analysis after our main variable, the CGPPE, was distributed. This method of gathering data inherently limits investigation into HCW outlooks before CGPPE supplies were acquired and whether the responses to this survey would significantly change had the survey also been offered prior to CGPPE distribution. Limitations also exist regarding whether respondents were informed of the respective percentages of commercial and community-generated PPE they received. Much of the CGPPE distributed through these hospitals was routed through administration rather than hand delivered to HCWs by donors. This may skew the results in regard to whether respondents felt their administrations and communities supported their safety and well-being during the COVID pandemic. Some respondents also forewent answering the survey in its entirety and questions closer to the bottom of the survey showed a decline in number of responses as a result. The maximum drop in respondents for any question was 17%, which indicates that roughly 82% of all survey initiates completed every question in the survey.

## Data Availability Statement

The raw data supporting the conclusions of this article will be made available by the authors, without undue reservation.

## Ethics Statement

The studies involving human participants were reviewed and approved by Alabama College of Osteopathic Medicine IRB. The patients/participants provided their written informed consent to participate in this study.

## Author Contributions

The following authors were responsible for the original design of the study including outline and drafting of the original manuscript: BB, JO, JM, AV, and MC. The primary author: BB. The following authors were responsible for data gathering, statistical analysis, and authorship of the methods and results section: CH, LF, JG, and BB. The following authors were in charge of final revision, final restructuring of the original manuscript, and article approval: BB, TH, WG, AV, and MC. All authors contributed to the article and approved the submitted version.

## Conflict of Interest

The authors declare that the research was conducted in the absence of any commercial or financial relationships that could be construed as a potential conflict of interest.

## Publisher's Note

All claims expressed in this article are solely those of the authors and do not necessarily represent those of their affiliated organizations, or those of the publisher, the editors and the reviewers. Any product that may be evaluated in this article, or claim that may be made by its manufacturer, is not guaranteed or endorsed by the publisher.

## Appendix

1. What is your age range?
  - 19–25
  - 25–35
  - 35–50
  - 50+
2. What care setting are you in?
  - Hospital
  - Physician Independent Clinic/Office
  - Dental Clinic/Office
  - Home Health/Assisted Living/Extended Care Facility
3. If you work in a hospital, what department?
  - ER
  - Non-ICU/Med Surg
  - Outpatient
  - NICU
  - ICU/CCU
  - Labor and Delivery
4. What is your title?
  - Healthcare Administration
  - Physician
  - PA/FNP
  - BSN/RN/LPN
  - RT
  - PT/OT/Speech Therapy
  - Support Associate (Reception, CNA, Med Tech, Xray Tech, etc)
  - Other: Please specify
5. What PPE did your organization provide prior to community manufactured PPE supply? (Check all that apply)
  - N95 Masks
  - Shields
  - Sterile Gowns
  - Surgical Masks
  - Other (Please Specify)
6. Have there been changes in the type of PPE supplied by your organization since the start of the COVID crisis?
  - Yes
  - No
7. Have there been changes in the volume/amount of PPE supplied by your organization since the start of the COVID crisis?
  - Yes
  - No
8. Have they Decreased/Increased?
  - Increased
  - Decreased
    COMMUNITY GENERATED PPE (CGPPE) includes 3d printed face shields, hand sewn masks, and ear savers (etc) that were given to your facility.
    (The following questions were given the likert scale options as follows: Strongly Agree, Agree, Neutral, disagree, Strongly Disagree.)
9. Does wearing the CGPPE provide you with the perception of added protection?
10. Are you more likely to support the use/production of CGPPE because they are made locally?
11. I personally feel that my administration supported my well-being and safety during the COVID pandemic.
12. I personally feel that my community supported my well-being and safety during the COVID Pandemic.
13. Wearing CGPPE improved my outlook toward working during the pandemic.
14. Using CGPPE increased my desire to work during the time of the Pandemic.
15. I would accept and use CGPPE in a future situation where there is an equipment shortage.
